# Obeticholic acid improves fetal bile acid profile in a mouse model of gestational hypercholanemia

**DOI:** 10.1152/ajpgi.00126.2020

**Published:** 2020-06-29

**Authors:** Vanessa Pataia, Saraid McIlvride, Georgia Papacleovoulou, Caroline Ovadia, Julie A. K. McDonald, Annika Wahlström, Eugène Jansen, Luciano Adorini, David Shapiro, Julian R. Marchesi, Hanns-Ulrich Marschall, Catherine Williamson

**Affiliations:** ^1^Department of Women and Children’s Health, King’s College London, London, United Kingdom; ^2^MRC Centre for Molecular Bacteriology and Infection, Imperial College London, London, United Kingdom; ^3^Department of Molecular and Clinical Medicine/Wallenberg Laboratory, Sahlgrenska Academy, University of Gothenburg, Gothenburg, Sweden; ^4^Centre for Health Protection, National Institute for Public Health and the Environment, Bilthoven, The Netherlands; ^5^Intercept Pharmaceuticals, San Diego, California; ^6^Department of Metabolism, Digestion and Reproduction, Imperial College London, London, United Kingdom; ^7^School of Biosciences, Cardiff University, Cardiff, United Kingdom

**Keywords:** bile acids, FXR, intrahepatic cholestasis of pregnancy

## Abstract

Intrahepatic cholestasis of pregnancy (ICP) is characterized by elevated maternal circulating bile acid levels and associated dyslipidemia. ICP leads to accumulation of bile acids in the fetal compartment, and the elevated bile acid concentrations are associated with an increased risk of adverse fetal outcomes. The farnesoid X receptor agonist obeticholic acid (OCA) is efficient in the treatment of cholestatic conditions such as primary biliary cholangitis. We hypothesized that OCA administration during hypercholanemic pregnancy will improve maternal and fetal bile acid and lipid profiles. Female C57BL/6J mice were fed either a normal chow diet, a 0.5% cholic acid (CA)-supplemented diet, a 0.03% OCA-supplemented diet, or a 0.5% CA + 0.03% OCA-supplemented diet for 1 wk before mating and throughout pregnancy until euthanization on *day 18*. The effects of CA and OCA feeding on maternal and fetal morphometry, bile acid and lipid levels, and cecal microbiota were investigated. OCA administration during gestation did not alter the maternal or fetal body weight or organ morphometry. OCA treatment during hypercholanemic pregnancy reduced bile acid levels in the fetal compartment. However, fetal dyslipidemia was not reversed, and OCA did not impact maternal bile acid levels or dyslipidemia. In conclusion, OCA administration during gestation had no apparent detrimental impact on maternal or fetal morphometry and improved fetal hypercholanemia. Because high serum bile acid concentrations in ICP are associated with increased rates of adverse fetal outcomes, further investigations into the potential use of OCA during cholestatic gestation are warranted.

**NEW & NOTEWORTHY** We used a mouse model of gestational hypercholanemia to investigate the use of obeticholic acid (OCA), a potent FXR agonist, as a treatment for the hypercholanemia of intrahepatic cholestasis of pregnancy (ICP). The results demonstrate that OCA can improve the fetal bile acid profile. This is relevant not only to women with ICP but also for women who become pregnant while receiving OCA treatment for other conditions such as primary biliary cholangitis and nonalcoholic steatohepatitis.

## Graphical abstract

**Figure:**
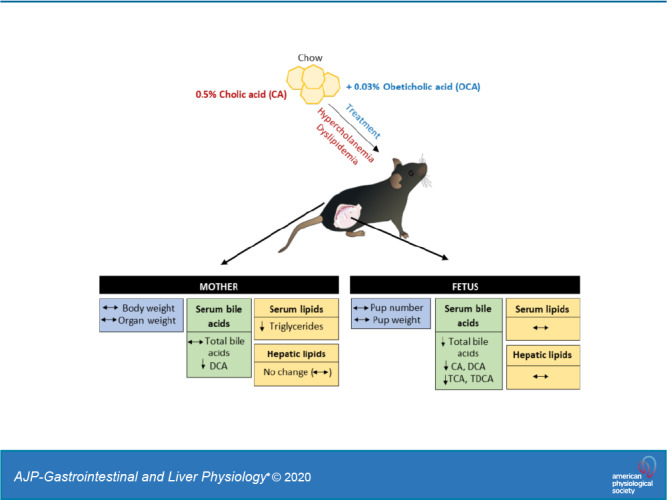

## INTRODUCTION

Intrahepatic cholestasis of pregnancy (ICP) is a cholestatic condition that affects 0.4–2.2% of pregnancies in North America and Western Europe but is more common in Chile and Bolivia, where it can affect 1.5–4% of pregnancies (11, 13, 44). ICP typically presents from 30 wk of gestation, and the main symptom is persistent generalized itch. Diagnosis is made in women with an elevation of serum bile acids. ICP is associated with maternal dyslipidemia (12, 27) and increased risk of gestational diabetes mellitus (26, 27, 49). The most common treatment for ICP is ursodeoxycholic acid (UDCA) administration, but not all patients respond (8, 9, 18), and a recent trial revealed no benefit for adverse perinatal outcomes (8).

The adverse fetal outcomes that occur in ICP include preterm birth, fetal hypoxia, meconium-stained amniotic fluid, stillbirth, and prolonged admission to the neonatal unit (19). Maternal bile acid levels have been reported to be positively correlated to fetal bile acid levels, and incremental rises in maternal serum bile acids >40 µmol/L are associated with higher risk of adverse fetal outcomes (7, 9, 21). The fetal lipid profile has also been shown to be affected by maternal cholestasis, with increased cholesterol accumulation in the fetal liver and placenta in a mouse model of gestational cholestasis and in the umbilical cord of neonates exposed to maternal ICP (41).

It has previously been described that during normal pregnancy the activity of farnesoid X receptor (FXR), the master nuclear receptor regulating bile acid homeostasis, is decreased, allowing for a maternal procholestatic profile even during normal gestation (31, 33, 39). However, it is thought that in ICP the combination of genetic susceptibility, elevated reproductive hormones, and environmental factors may lead to an exacerbation of the procholestatic profile found in pregnancy and result in a pathological rise of bile acid levels (17).

In recent years, synthetic FXR agonists have been developed. In particular, the semisynthetic bile acid obeticholic acid (OCA) has more than 100 times higher affinity for FXR than its most potent natural ligand, chenodeoxycholic acid (CDCA), and has been shown to promote bile acid efflux and reduce bile acid synthesis (51). Clinical trials of OCA have shown promising results for the treatment of primary biliary cholangitis (PBC) and nonalcoholic steatohepatitis (NASH) (2).

In this study, we used a previously established model of 0.5% cholic acid (CA) feeding in pregnancy to mimic the hypercholanemia of ICP (32, 41). Because of the key role of FXR in bile acid synthesis, transport, and excretion, as well as regulation of lipid metabolism, we hypothesized that activation of FXR by OCA could improve maternal and fetal hypercholanemia and dyslipidemia.

## MATERIALS AND METHODS

### Animal experiments.

Six- to eight-week-old C57BL/6J mice were purchased from Envigo and allowed to acclimatize for 1 wk before any experimental procedures were carried out. All mice were kept on a 12:12-h light-dark cycle with access to food and water ad libitum. All procedures were approved by the Animal Welfare and Ethical Review Body at King’s College London and carried out according to the UK Animals (Scientific Procedures) Act 1986. All diets were supplied by Special Diet Services.

We have previously shown that cholic acid (CA) feeding can induce maternal hypercholanemia in mice (32, 41). Female mice were assigned to either standard maintenance or breeding diet (CRM), referred to as normal chow diet (NC), a 0.5% CA-supplemented CRM diet, a 0.03% obeticholic acid (OCA; Intercept Pharmaceuticals)-supplemented CRM diet, or a 0.5% CA + 0.03% OCA (CA + OCA)-supplemented CRM diet 1 wk before mating and maintained on their assigned diet for the duration of the experimental procedures. The dose of OCA was selected based on previously published literature (4) and was equivalent to ∼42 mg·kg^−1^·day^−1^. Females were mated to control males and checked daily for the presence of a copulatory plug. The day of identification of the copulatory plug was considered *day 1* of pregnancy (D1). Body weight of pregnant females was measured on *days 7* (D7), *14* (D14), and *18* (D18) of pregnancy. On D18, females were fasted for 4 h and euthanized by CO_2_ inhalation. Maternal and fetal sera were collected, and pup number per litter was assessed. Maternal liver, subcutaneous white adipose tissue (sWAT), gonadal white adipose tissue (gWAT), brown adipose tissue (BAT), and fetal and placental weight were measured. Maternal liver, terminal ileum, fetal liver, and placenta were collected and snap-frozen. Nonpregnant control female mice were maintained on the same diets as pregnant females for an equivalent length of time and were assessed for the same parameters.

### Gene expression studies.

Total RNA was extracted from frozen tissue samples using the RNeasy Mini kit (Qiagen) according to the manufacturer’s guidance. After RNA extraction, 1 μg of total RNA was reverse transcribed using SuperScript II Reverse Transcriptase (Invitrogen). RNaseOUT Recombinant Ribonuclease Inhibitor (Invitrogen) was used as an RNase inhibition step. Assessment of the expression of target genes of interest was assessed using quantitative RT-PCR with a ViiA 7 Real Time PCR System (Thermo Fisher Scientific) by adding cDNA in duplicate to a 384-well plate, followed by a reaction mix of 1× SYBR Green Jumpstart Readymix (Sigma-Aldrich) and 1 μM of forward/reverse primers. The housekeeping gene *cyclophilin b* was used as an internal reference for quantification of relative gene expression. Primer sequences of genes of interest are provided in Supplemental Table S1 (Supplemental Material for this article can be found online at https://doi.org/10.6084/m9.figshare.12497714).

### Serum and tissue lipid quantification.

Serum and tissue lipid content were extracted and measured as previously described (38). In brief, frozen tissues of interest were homogenized in Hanks’ Balanced Salt Solution using a TissueLyser II (Qiagen) system. Samples were then centrifuged at 12,000 rpm for 15 min at 4°C (Rotina 420R Benchtop Centrifuge, Hettich, Germany). The supernatant was discarded. The pellet was resuspended in 500 μL of lysis buffer containing 0.125 M potassium phosphate, 1 mM EDTA, and 0.1% Triton X-100 at pH 7.4. Samples were sonicated at 4°C for 8 min in a Bioruptor Plus (4 cycles of sonication for 30 s, followed by 4 cycles of resting for 30 s). Samples were subsequently centrifuged at 10,000 rpm for 15 min at 4°C. Total cholesterol, LDL cholesterol, HDL cholesterol, triglycerides (TGs), free fatty acids (FFAs), and total protein were measured in plasma and tissue extracts with an Unicel DxC 800 autoanalyzer (Beckman-Coulter) using dedicated kits, with the exception of FFAs which were measured using a kit from Wako Diagnostics (Germany). The measurements in the tissue extracts were normalized with the protein content of each individual tissue sample.

### Serum and cecal bile acid quantification.

Measurements of serum and cecal bile acids were performed on an ultraperformance liquid chromatography Alliance 2695 system coupled to a Xevo TQ mass spectrometer using a SunFire C18 column, as previously described (1, 45). Analytes were detected using selected ion monitoring and quantified against deuterium-labeled internal standards. Quantification was achieved by comparison of peak height of molecular anions or negative daughter to the peak height of the deuterated internal standards.

### 16S rRNA gene sequencing analysis.

Cecal samples were homogenized, and DNA was extracted using the QIAamp Fast DNA Stool Mini Kit (Qiagen) according to the manufacturers’ protocol. Sample libraries were prepared as previously described (28) using the V1-V2 primers (35). An Illumina MiSeq platform was used to perform the sequencing with the MiSeq Reagent Kit version 3 and paired-end 300-bp chemistry (Illumina, Inc.). Mothur software (version 1.35.1; www.mothur.org) was used for data analysis, following the MiSeq SOP Pipeline (47). The Silva bacterial database (www.arb-silva.de/) was used for sequence alignments, and sequences were classified according to the RDP database reference sequence files using the Wang method (16). The UniFrac weighted distance matrix created by Mothur was used to produce nonmetric multidimensional scaling (NMDS) plots and PERMANOVA (permutational multivariate analysis of variance) *P* values, and analysis was carried out using the Vegan library (6) within the R statistical software (www.r-project.org). Bacterial relative abundance was expressed as extended error bar plots using the Statistical Analysis of Metagenomic Profiles software package and analyzed by White’s nonparametric *t* test with Benjamini-Hochberg False Discovery Rate (FDR). The α-diversity (Shannon diversity index, H′) was calculated using Mothur, and Tukey’s Honestly Significant Difference test was performed using IBM SPSS Statistics Software version 23. *P* and *q* values of 0.05 were considered to be significant.

### Statistical analysis.

All values are shown as means ± SE. Statistical analysis was performed using GraphPad Prism 7 software. One-way ANOVA followed by a Newman-Keuls post hoc test was used, with a significance cutoff of *P* ≤ 0.05. Statistical analysis of 16S rRNA gene sequencing data is detailed in the relevant section above.

## RESULTS

### OCA administration during pregnancy does not negatively impact maternal or fetal morphometry.

**Fig. 1.**
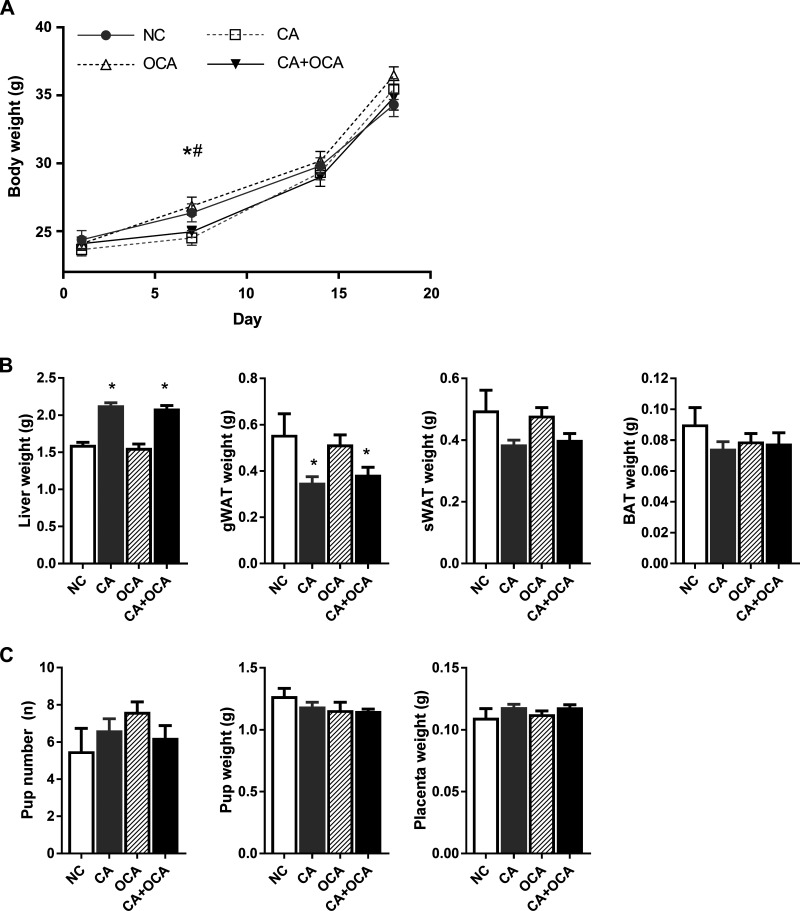
Effects of hypercholanemia and obeticholic acid (OCA) treatment during pregnancy on body and organ morphometry. *A*: body weight of pregnant females on *days 1* (D1), *7* (D7), *14* (D14), and *18* (D18). #*P* ≤ 0.05 for cholic acid (CA) vs. OCA; *P ≤ 0.05 for CA + OCA vs. OCA groups. *B*: weight of liver, gonadal white adipose tissue (gWAT), subcutaneous white adipose tissue (sWAT), and brown adipose tissue (BAT) of pregnant females on D18. *C*: pup number, pup weight, and placenta weight of D18 fetuses. *P ≤ 0.05 in comparisons vs. normal chow diet (NC) and OCA groups. Data are presented as means ± SE; *n* = 6–9 mice.

We first aimed to establish the effect of hypercholanemia and OCA supplementation during pregnancy on body weight and organ morphometry. During pregnancy, no body weight differences were seen between groups, except on D7 when CA and CA + OCA-fed females were significantly lighter than OCA-fed females (Fig. 1*A*). Although no body weight differences were registered on D18 gestation, pregnant females fed a CA diet had increased liver weight and decreased gWAT weight regardless of OCA cofeeding (Fig. 1*B*). A trend for decreased sWAT weight was also seen in pregnant CA and CA + OCA groups (Fig. 1*B*). OCA supplementation alone did not affect body weight or organ morphometry (Fig. 1*B*).

Despite the changes in maternal liver and gWAT morphometry in the CA and CA + OCA-fed groups, no changes in pup number, pup weight, or placental weight were registered (Fig. 1*C*).

Outside of pregnancy, both CA and CA + OCA nonpregnant females were lighter than NC- and OCA-supplemented females on D18 (Supplemental Fig. S1A). This weight difference likely reflected a decrease in gWAT, sWAT, and BAT depot weight despite an increase in liver weight (Supplemental Fig. S1B).

These results demonstrate that OCA administration either alone or to hypercholanemic pregnant females did not negatively impact maternal or fetal body or organ morphometry.

### OCA administration during hypercholanemic pregnancy reduces fetal hypercholanemia.

**Fig. 2.**
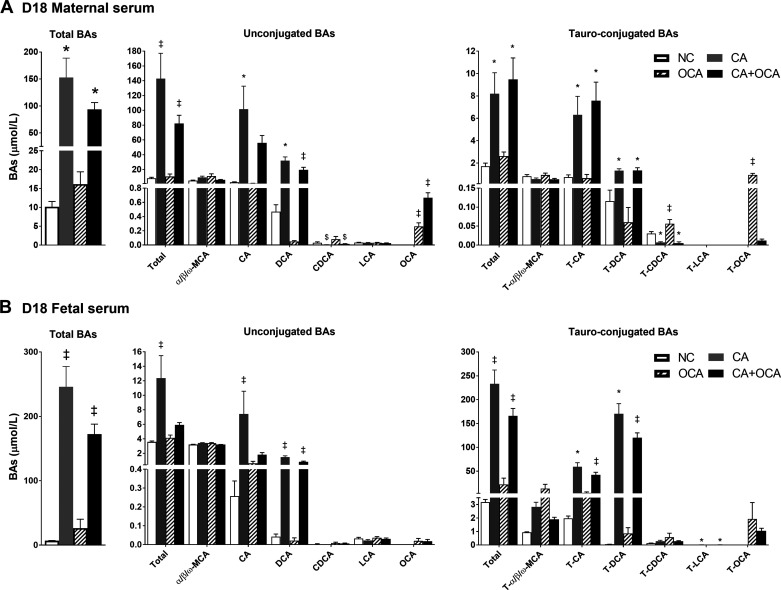
Effects of hypercholanemia and obeticholic acid (OCA) treatment during pregnancy on serum bile acid (BA) profile. *A*: serum total BAs, unconjugated BA, and taurine-conjugated BA levels in *day 18* (D18) pregnant females; *n* = 6 mice /group. *B*: serum total BA, unconjugated BA, and taurine-conjugated BA levels in D18 fetuses; *n* = 4–6 mice /group. *P ≤ 0.05 in comparisons vs. normal chow diet (NC) and OCA groups; ‡*P* ≤ 0.05 in comparisons vs. all groups; $*P* ≤ 0.05 in comparisons vs. OCA; †*P* ≤ 0.05 in comparisons vs. NC and CA groups. Data are presented as means ± SE.

We next investigated whether OCA administration ameliorated the maternal and fetal bile acid profiles during hypercholanemic gestation. In pregnant females, CA feeding led to a significant increase in total serum bile acid levels, CA, deoxycholic acid (DCA), taurocholic acid (TCA), and taurodeoxycholic acid (TDCA) compared with NC controls, confirming that CA-feeding induces maternal hypercholanemia, as has previously been described (32, 41). CA + OCA cosupplementation did not ameliorate total serum bile acid levels, although total unconjugated bile acids were significantly reduced compared with CA alone due to changes in CA (*P* > 0.05) and DCA (*P* ≤ 0.05) (Fig. 2*A*).

In nonpregnant females, total bile acids and DCA, TCA, and TDCA levels were significantly elevated by CA feeding and were not reduced by CA + OCA cofeeding (Supplemental Fig. S2).

In the fetal compartment, maternal hypercholanemia led to a significant rise in fetal serum total bile acids (Fig. 2*B*). However, total serum bile acid levels were 29.9% lower in fetuses from mothers fed a CA+OCA diet compared with CA alone, although still higher than NC controls (Fig. 2*B*). This was due to decreased concentrations of DCA, TCA, TDCA, and, in particular, CA (Fig. 2*B*). Maternal OCA feeding alone did not change fetal bile acid concentrations, although the presence of OCA and T-OCA in the fetal circulation suggests that OCA is able to cross the placenta (Fig. 2*B*).

Overall, OCA administration to hypercholanemic females did not significantly ameliorate maternal hypercholanemia but improved the fetal bile acid profile.

### OCA administration alone reduces cecal bile acid levels.

**Fig. 3.**
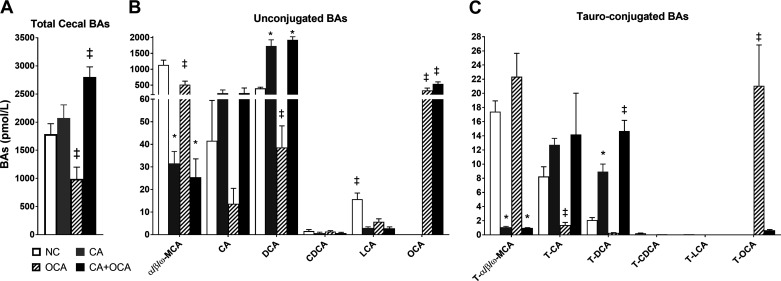
Effects of hypercholanemia and obeticholic acid (OCA) treatment during pregnancy on cecal bile acid profile. Bile acid (BA) levels in cecum of *day 18* (D18) pregnant females. *A*: total cecal BAs. *B*: unconjugated BAs. *C*: taurine-conjugated bile acids. Data are presented as means ± SE; *n* = 6–9 mice. ‡*P* ≤ 0.05 in comparisons vs. all groups; *P ≤ 0.05 in comparisons vs. normal chow diet (NC) and OCA groups.

Cecal bile acid concentrations were also measured. As expected in the cecum, bile acids were largely unconjugated (Fig. 3). Total cecal bile acid levels were significantly increased in mice fed CA + OCA compared with CA alone; however, this was largely due to enrichment with OCA and also with DCA that increased in the CA-fed group (Fig. 3, *A* and *B*). Muricholic acids levels were markedly reduced in both CA and CA + OCA groups (Fig. 3, *B* and *C*). OCA administration alone significantly reduced total cecal bile acid levels compared with all other groups, which was due to an overall reduction in bile acids (Fig. 3*A*). Interestingly, as seen in the serum, T-OCA levels were significantly lower in CA + OCA cofed mice compared with females supplemented with OCA only, whereas OCA levels were increased (Fig. 3, *B* and *C*).

### Bile acid supplementation impacts the cecal microbiome’s microbiota composition.

**Fig. 4.**
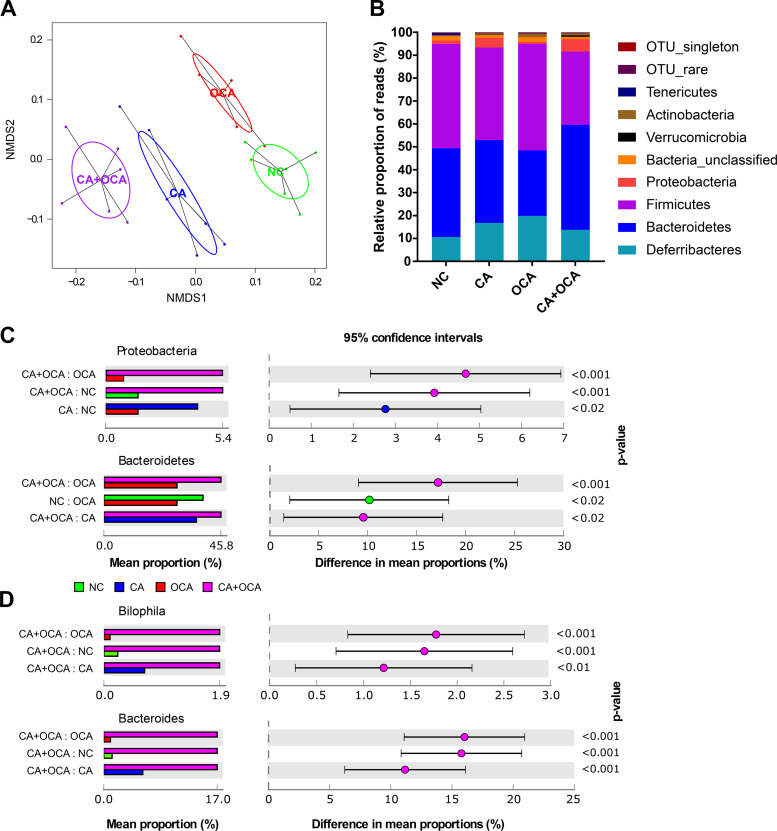
Changes in cecal microbiota in pregnant mice measured by 16S rRNA gene sequencing. *A*: nonmetric multidimensional scaling (NMDS) plot showing differences in bacterial community structure based on the weighted UniFrac distance metric. For *P* values, see Supplemental Table S2. *B*: changes in relative proportion of reads at phylum level. *C* and *D*: significant changes in the average relative proportion of sequences assigned to each taxa for each dietary group at phylum (*C*) and genus level (*D*). Data presented as extended error bar plots showing *P* value, effect size, and confidence interval for each taxa. Analyzed by Kruskal-Wallis *H* test with Benjamini-Hochberg false discovery rate; *n* = 6 mice/group. CA, cholic acid; NC, normal chow diet; OCA, obeticholic acid.

Conversion of primary to secondary bile acids, as well as bile acid deconjugation, is performed by intestinal bacteria. Because changes in bile-metabolizing bacteria will affect the host bile acid pool, the cecal bacterial community was investigated by 16S rRNA gene sequencing. Nonmetric multidimensional scaling (NMDS) analysis of weights UniFrac distances, which shows how the microbial communities vary between the groups, demonstrates significant differences between all the dietary groups in pregnant mice (Fig. 4*A* and Supplemental Table S2). OCA supplementation alone was the least different from NC, with CA and then CA + OCA being more dissimilar. Differences in the relative proportion of phyla were observed between pregnant groups (Fig. 4*B*); specifically, both CA feeding and CA + OCA cofeeding significantly increased the relative abundance of *Proteobacteria* in the cecum of pregnant mice compared with NC groups (Fig. 4*C*). OCA feeding alone did not significantly impact *Proteobacteria*, but the relative abundance of *Bacteroidetes* was significantly decreased in pregnant females (Fig. 4*C*). Significant changes were also observed at genus level, with an increase in the relative proportion of *Bilophila* and *Bacteroides* in CA + OCA-fed mice compared with all other groups (Fig. 4*D*). This was reinforced by correlation analysis between microbiota and bile acid concentrations in the cecum, which showed that *Proteobacteria* and *Bacteroidetes* positively correlated with OCA and negatively with T-OCA concentrations (Supplemental Fig. S3A). Alpha diversity (Shannon diversity index) plots showed that CA supplementation alone or cofed with OCA resulted in decreased bacterial diversity (Supplemental Fig. S3B). Pregnancy caused a significant increase in an unclassified class of *Bacteroidetes* in NC controls (Supplemental Fig. S3C). In nonpregnant mice, NMDS analysis and alpha diversity plots were similar to pregnant mice (Supplemental Fig. S4, A and B). However, changes between the dietary groups differed at phylum level; in particular, significant differences were observed in *Bacteroidetes*, *Firmicutes*, and *Proteobacteria* (Supplementary Fig. S4C).

### OCA administration represses maternal hepatic Cyp7a1 expression via intestinal FXR.

To further assess the effects of hypercholanemia and OCA administration on bile acid homeostasis during pregnancy, the expression of key genes for bile acid homeostasis in the liver and terminal ileum was investigated.

**Fig. 5.**
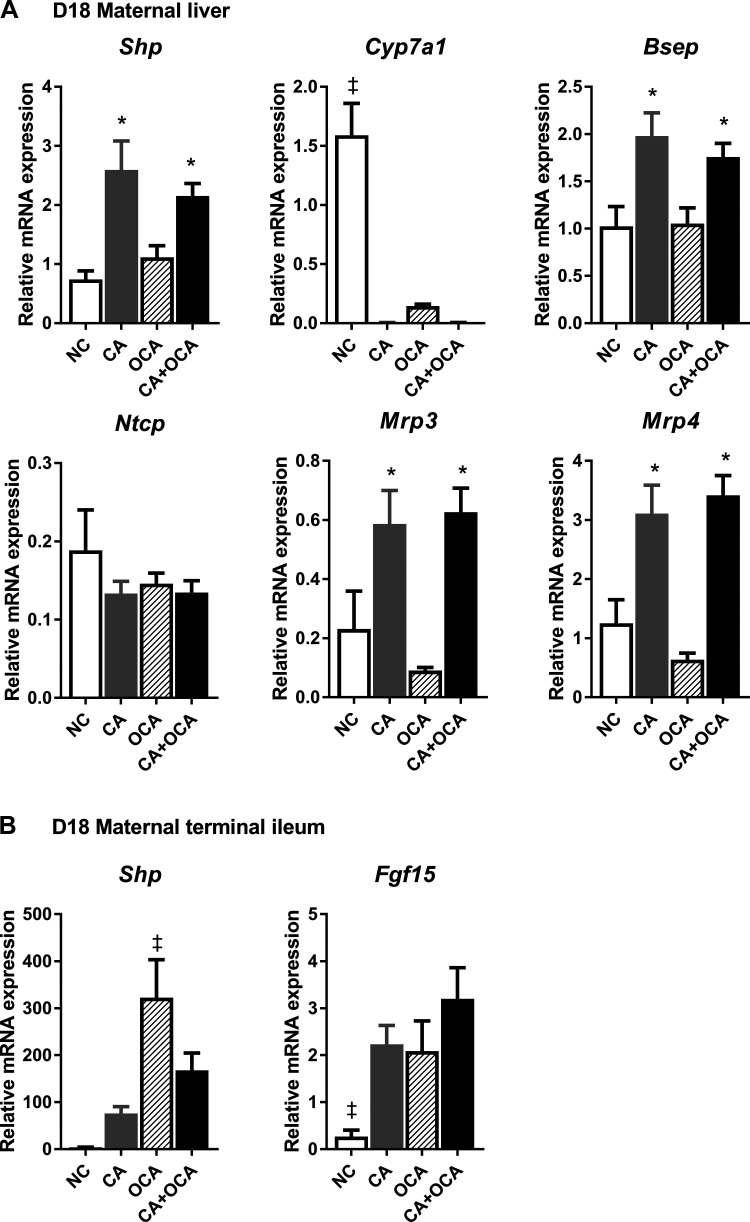
Expression of key bile acid homeostasis genes in pregnant females. *A*: mRNA expression of genes regulating bile acid synthesis and transport in the liver. *B*: mRNA expression of genes regulating bile acid synthesis and transport in the terminal ileum. Data are presented as means ± SE; *n* = 4–6 mice. *P ≤ 0.05 in comparisons vs. normal chow diet (NC) and obeticholic acid (OCA groups); ‡*P* ≤ 0.05 in comparisons vs. all groups. CA, cholic acid; D18, *day 18* pregnant.

The hepatic FXR target *Shp* was significantly upregulated in pregnant females fed a CA or a CA + OCA diet, and this change was concomitant with the repression of hepatic *Cyp7a1* (Fig. 5*A*). Both CA and CA + OCA diet increased the hepatic expression of the bile acid transporters *Bsep*, *Mrp3*, and *Mrp4* in pregnant females (Fig. 5*A*). Whereas OCA supplementation alone did not induce significant hepatic *Shp* upregulation, *Cyp7a1* expression was significantly decreased in D18 pregnant females (Fig. 5*A*). In parallel, intestinal *Shp* expression was upregulated in OCA-fed females, and intestinal *Fgf15* expression was significantly increased by maternal CA, OCA, and CA + OCA supplementation (Fig. 5*B*).

In nonpregnant females, relative mRNA expression followed a very similar pattern to pregnant mice (Supplemental Fig. S5, A and B). Of note is that lower hepatic gene expression of several FXR targets was observed in pregnant mice compared with nonpregnant mice, regardless of diet (Table 1). Expression of FXR targets in the terminal ileum was similarly affected by pregnancy. In pregnant CA-fed females, *Shp* and *Fgf15* expression were lower than outside pregnancy (Table 2). *Shp* expression levels were also lower in CA + OCA-fed pregnant females compared with nonpregnant (Table 2).

**Table 1.** Effect of pregnancy on hepatic mRNA expression of key bile acid homeostasis genes

	NC		CA		OCA		CA + OCA	
	NP	P	NP	P	NP	P	NP	P
*Shp*	2.06 ± 0.35	0.74 ± 0.14*	3.36 ± 0.63	2.59 ± 0.49	1.78 ± 0.39	1.12 ± 0.19	3.46 ± 0.49	2.15 ± 0.21*
*Cyp7a1*	1.37 ± 0.37	1.59 ± 0.27	0.004 ± 0.001	0.004 ± 0.002	0.66 ± 0.21	0.14 ± 0.02*	0.006 ± 0.0023	0.005 ± 0.001
*Ntcp*	0.77 ± 0.21	0.19 ± 0.05*	0.29 ± 0.06*	0.13 ± 0.02*	0.36 ± 0.03	0.15 ± 0.01*	0.28 ± 0.05	0.14 ± 0.01*
*Bsep*	1.74 ± 0.38	1.02 ± 0.21	2.78 ± 0.36	1.98 ± 0.25	2.05 ± 0.37	1.05 ± 0.17*	2.62 ± 0.34	1.76 ± 0.15*
*Mrp3*	1.31 ± 0.12	0.23 ± 0.13*	2.68 ± 0.41	0.59 ± 0.11*	1.07 ± 0.17	0.09 ± 0.01*	2.28 ± 0.29	0.63 ± 0.08*
*Mrp4*	1.17 ± 0.12	1.25 ± 0.40	4.87 ± 0.21	3.11 ± 0.48*	1.56 ± 0.28	0.63 ± 0.11*	4.58 ± 0.61	3.412 ± 0.34

Data are presented as means ± SE; *n* = 3–6 mice. CA, cholic acid; NC, normal chow diet; NP, nonpregnant; OCA, obeticholic acid; P, pregnant. Relative mRNA expression of target genes in NP and P females fed the same diet.

* *P* ≤ 0.05 vs. NP females fed the same diet.

**Table 2.** Effect of pregnancy on mRNA expression of key bile acid homeostasis genes in the terminal ileum

	NC		CA		OCA		CA + OCA	
	NP	P	NP	P	NP	P	NP	P
*Shp*	3.17 ± 1.96	3.93 ± 0.36	229.70 ± 57.30	76.18 ± 14.53*	670.4 ± 211.6	322.50 ± 80.76	398.90 ± 73.87	167.70 ± 36.85*
*Fgfr15*	1.47 ± 0.57	0.27 ± 0.14	4.29 ± 0.27	2.23 ± 0.40*	2.88 ± 0.55	2.08 ± 0.64	4.10 ± 0.62	3.20 ± 0.66

Data are presented as means ± SE; *n* = 3–6 mice. CA, cholic acid; NC, normal chow diet; NP, nonpregnant; OCA, obeticholic acid; P, pregnant. Relative mRNA expression of target genes in NP and P females fed the same diet.

* *P* ≤ 0.05 vs. NP females fed the same diet.

Overall, we conclude that despite decreased expression of FXR target genes during pregnancy, activation of intestinal rather than hepatic FXR can mediate OCA-induced suppression of hepatic *Cyp7a1* expression.

### Maternal OCA administration represses fetal hepatic Cyp7a1 expression.

**Fig. 6.**
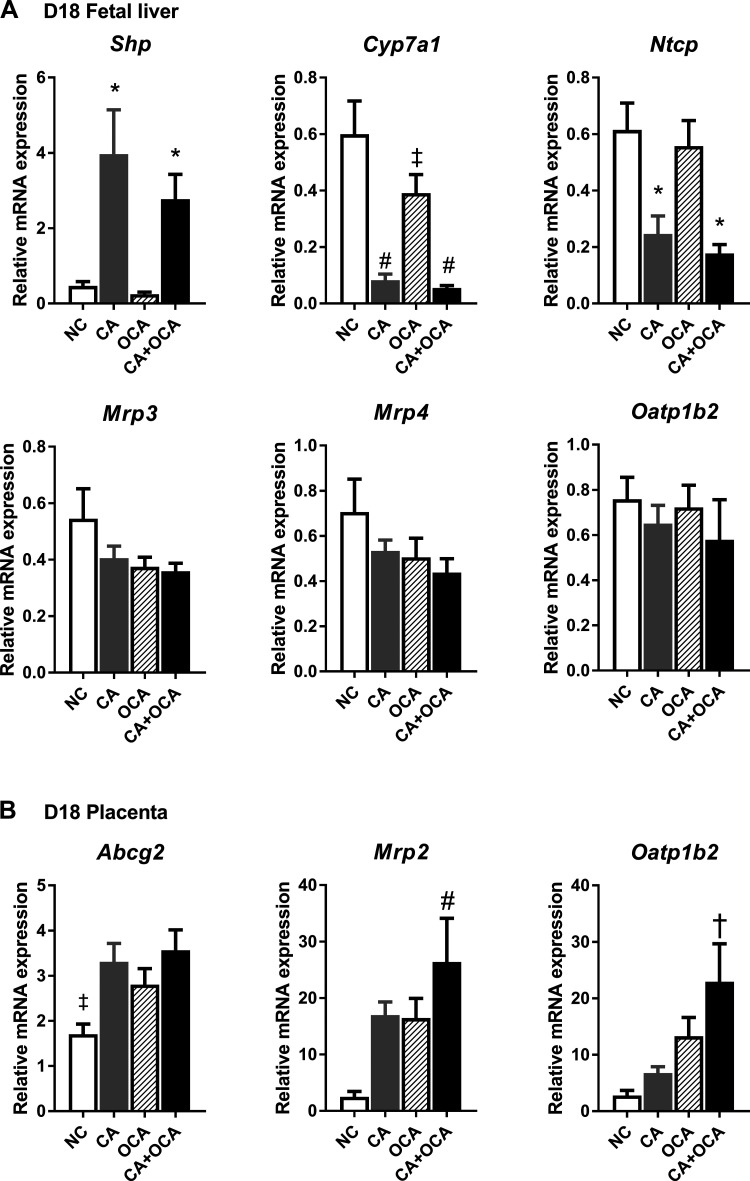
Expression of key bile acid homeostasis genes in the fetoplacental unit. *A*: mRNA expression of genes regulating bile acid synthesis and transport in the fetal liver. *B*: mRNA expression of genes regulating bile acid transport in the placenta. Data are presented as means ± SE; *n* = 5–6 mice. *P ≤ 0.05 in comparisons vs. normal chow diet (NC) and obeticholic acid (OCA) groups; #*P* ≤ 0.05 in comparisons vs. NC; ‡*P* ≤ 0.05 in comparisons vs. all groups; †*P* ≤ 0.05 in comparisons vs. NC and cholic acid (CA) groups. D18, *day 18* pregnant.

Given the decrease in fetal serum bile acid concentrations in maternal CA + OCA feeding groups, the expression of key bile acid homeostasis genes in the fetal liver and placenta were assessed. Maternal CA feeding alone or cosupplemented with OCA induced an upregulation of *Shp* expression and a concomitant reduction in *Cyp7a1* and *Ntcp* in the fetal liver (Fig. 6*A*). Of note is that whereas maternal OCA diet alone did not have an impact on fetal hepatic *Shp* expression, a significant downregulation of hepatic *Cyp7a1* expression was observed, albeit to a lesser extent than in groups with maternal CA supplementation (Fig. 6*A*). Maternal bile acid feeding did not have an impact on hepatic fetal *Mrp3*, *Mrp4*, or *Oatp1b2* expression (Fig. 6*A*).

As the placenta plays a crucial role in bile acid transport between maternal and fetal circulations, we further sought to determine whether maternal OCA administration had an impact on placental bile acid transporter gene expression. Interestingly, all maternal bile acid feeding groups showed a significant upregulation of *Abcg2* expression in the placenta (Fig. 6*B*). Moreover, maternal CA+OCA feeding increased placental *Mrp2* expression when compared against all other feeding groups, and *Oatp1b2* expression was increased compared with NC and CA groups (Fig. 6*B*). Overall, we conclude that OCA modulates the expression of *Cyp7a1* in the fetal liver and bile acid transporters in the placenta.

### OCA administration during hypercholanemic pregnancy does not reverse maternal dyslipidemia.

**Fig. 7.**
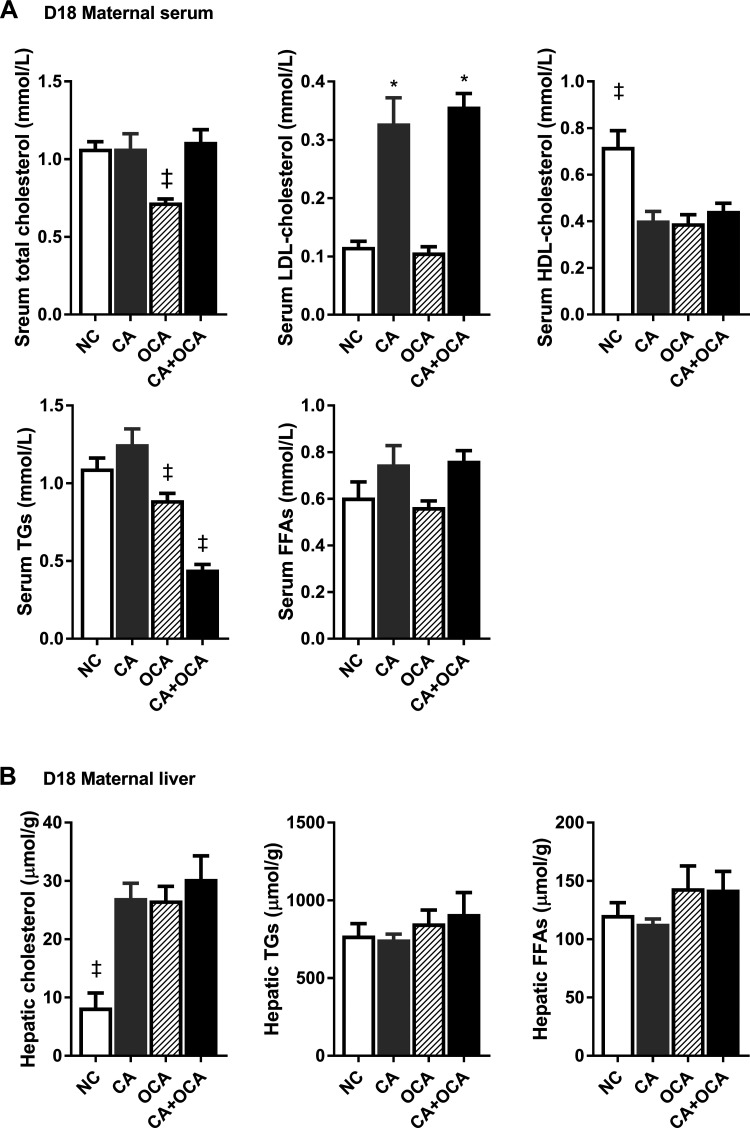
Effects of hypercholanemia and obeticholic acid (OCA) treatment during pregnancy on serum and hepatic lipid levels. *A*: serum lipid levels. *B*: hepatic lipid levels. Data are presented as means ± SE; *n* = 4–6 mice. ‡*P* ≤ 0.05 in comparisons vs. all groups; *P ≤ 0.05 in comparisons vs. normal chow diet (NC) and OCA groups. CA, cholic acid; D18, *day 18* pregnant; FFAs, free fatty acids; TGs, triglycerides.

Cholestasis is commonly accompanied by dyslipidemia. Hence, we next studied the effect of OCA administration during hypercholanemic pregnancy on maternal and fetal serum and hepatic lipid levels. No changes in total serum cholesterol levels were seen in pregnant CA or CA + OCA-supplemented groups (Fig. 7*A*). However, females exposed to a CA or CA + OCA diet had raised serum LDL cholesterol and decreased HDL cholesterol levels compared with NC females (Fig. 7*A*) also outside of pregnancy (Supplemental Fig. S6A). Conversely, OCA feeding resulted in decreased total serum cholesterol levels compared with NC controls, which was associated with a reduction in serum HDL cholesterol concentrations (Fig. 7*A*). Serum HDL cholesterol was also reduced in nonpregnant OCA-fed mice (Supplemental Fig. S6A). CA feeding did not alter serum triglyceride levels in pregnant females, but OCA diet reduced serum triglyceride levels, and a further decrease was observed in CA + OCA-fed females (Fig. 7*A*). In contrast, no significant changes were observed in serum triglyceride levels in nonpregnant females (Supplemental Fig. S6A).

In the liver, CA, OCA, and CA + OCA supplementation of pregnant females led to hepatic cholesterol accumulation compared with the NC control group (Fig. 7*B*). In nonpregnant females, hepatic cholesterol levels were significantly lower with OCA supplementation alone compared with CA and CA + OCA-fed mice (Supplemental Fig. S6B).

Taken together, these data led us to conclude that OCA administration does not ameliorate maternal dyslipidemia during hypercholanemic gestation.

### OCA administration during hypercholanemic pregnancy does not reverse fetal dyslipidemia.

**Fig. 8.**
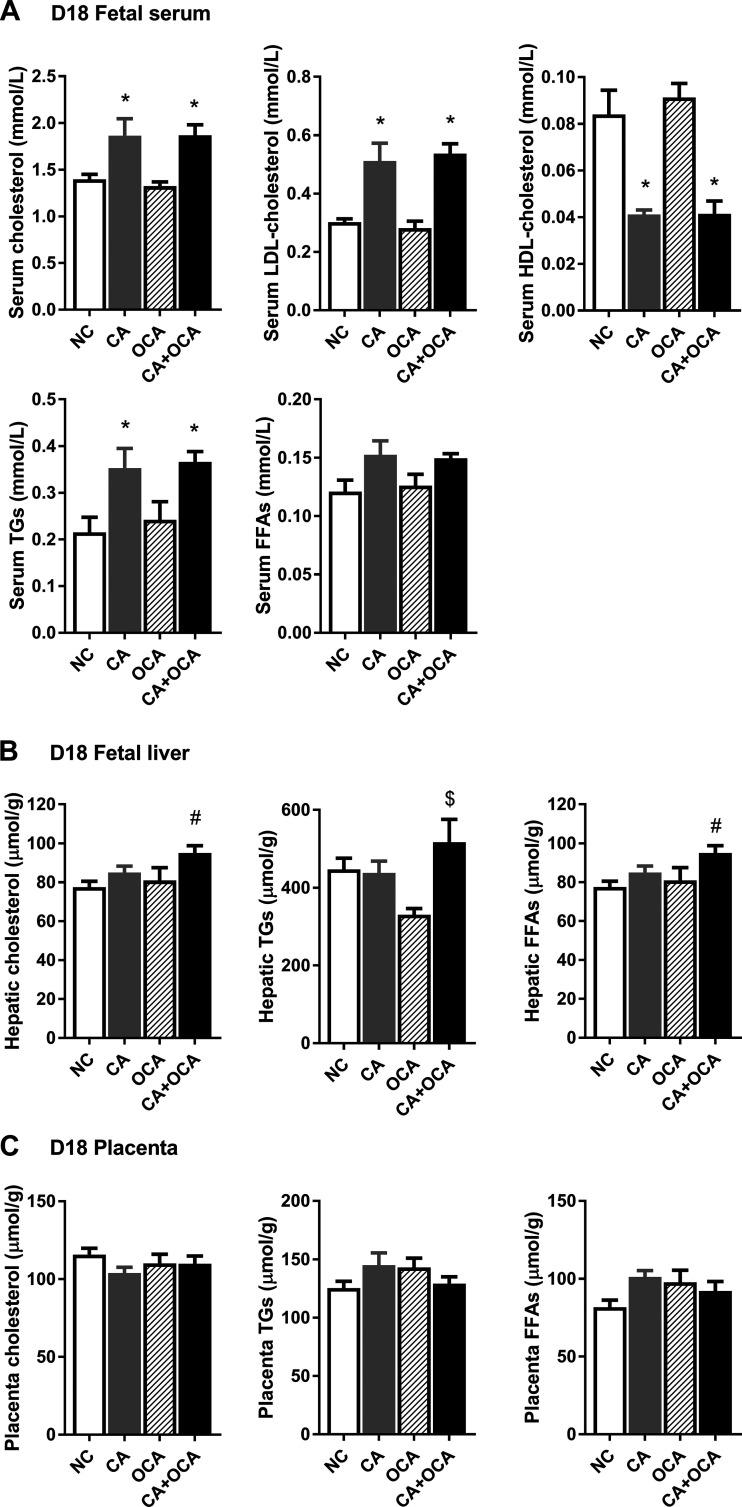
Effects of hypercholanemia and obeticholic acid (OCA) treatment on lipid levels in the fetoplacental unit. *A*: fetal serum lipid levels. *B*: fetal hepatic lipid levels. *C*: placental lipid levels. Data are presented as means ± SE; *n* = 4–6 mice. *P ≤ 0.05 in comparisons vs. normal chow diet (NC) and OCA groups; #*P* ≤ 0.05 in comparisons vs. NC; $*P* ≤ 0.05 in comparisons vs. OCA group. CA, cholic acid; FFAs, free fatty acids; TGs, triglycerides.

Because maternal dyslipidemia is commonly associated with fetal dyslipidemia, we next investigated the fetal lipid profile. Maternal CA feeding significantly increased fetal serum cholesterol levels, including LDL cholesterol, and this was not altered by maternal CA + OCA supplementation (Fig. 8*A*). In parallel, fetal serum HDL cholesterol concentrations were reduced in maternal CA and CA + OCA supplementation groups. Fetal circulating triglycerides were increased in fetuses from CA-fed mothers and were not improved by maternal CA + OCA feeding (Fig. 8*A*). Of note is that maternal OCA feeding alone had no effect on fetal total and LDL or HDL cholesterol levels or triglyceride and FFA concentrations (Fig. 8*A*).

Fetal hepatic cholesterol and FFA content were increased in fetuses from CA + OCA-fed mothers compared with NC mothers (Fig. 8*B*). However, maternal OCA diet alone did not affect fetal cholesterol and FFA accumulation in the liver (Fig. 8*B*). A trend for increased hepatic cholesterol and FFA was also observed in fetuses from CA-fed mothers compared with NC controls, although it did not reach statistical significance (Fig. 8*B*).

To assess a potential relationship between fetal and placental lipid levels, the placental lipid content on D18 of gestation was also evaluated. However, no significant changes in placental cholesterol, triglycerides, or FFA content were registered between different groups (Fig. 8*C*).

**Fig. 9.**
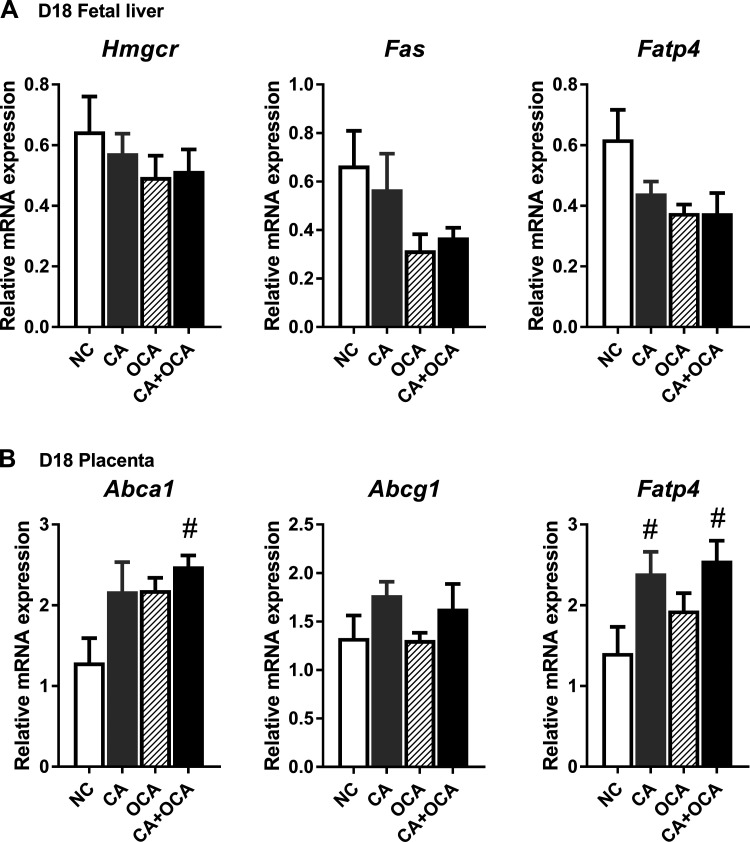
Effects of hypercholanemia and obeticholic acid (OCA) treatment on lipid homeostasis genes in the fetoplacental unit. *A*: expression of key hepatic lipid biosynthesis and transport genes in the fetal liver. *B*: placental expression of lipid transport genes. Data are presented as means ± SE; *n* = 4–6 mice. #*P* ≤ 0.05 in comparisons vs. normal chow diet (NC). CA, cholic acid.

We subsequently aimed to establish whether the changes in the fetal lipid profile on D18 of gestation were due to shifts in lipid de novo biosynthesis and transport in the fetal liver or placenta. Maternal bile acid feeding did not impact fetal hepatic *Hmgcr*, *Fas*, or *Fatp4* expression (Fig. 9*A*). However, maternal CA + OCA feeding led to a significant increase in placental expression of the cholesterol transporter *Abca1* compared with NC placentas (Fig. 9*B*). Interestingly, maternal CA and CA + OCA supplementation, but not maternal OCA alone, resulted in a significant increase in *Fatp4* placental expression compared with NC controls (Fig. 9*B*). Taken together, these data lead us to conclude that OCA administration does not ameliorate fetal dyslipidemia during hypercholanemic gestation.

## DISCUSSION

ICP is the commonest gestational liver disease and can lead to adverse fetal outcomes (19, 21, 40). Increased rates of stillbirth, spontaneous preterm birth, and meconium-stained amniotic fluid have been reported in pregnancies with high maternal serum concentrations of bile acids (19, 21, 40), likely related to fetal exposure to high bile acid concentrations (7). Although UDCA treatment of ICP has been shown to reduce maternal bile acid levels in some studies (23), it is not effective in all patients (8), and it does not return fetal bile acid levels to normal concentrations (20). The present study shows that OCA administration in a mouse model of hypercholanemia, as seen in ICP, is not detrimental to the mother or fetus and improves fetal hypercholanemia.

In our model, CA feeding led to significantly raised total bile acids in fetal serum. This was largely due to an increase in taurine-conjugated CA and DCA. Although the fetus synthesizes bile acids from early pregnancy onward, maternal bile acids can also cross the placenta and contribute to the fetal bile acid pool (29). Unconjugated and, at much lower levels, taurine-conjugated CA and DCA were also raised in the serum of CA-fed mothers. In the fetal compartment, DCA must be maternally derived since the fetus cannot synthesize secondary bile acids due to the absence of gut flora, and it is possible that CA is also being transferred from the mother. However, it is not known whether there is preferential transport of more hydrophilic taurine conjugates across the placenta or increased taurine conjugation occurring in the fetal liver. We have previously observed in humans that the ratio of conjugated to unconjugated bile acids is higher in umbilical cord blood than in maternal serum (20).

OCA treatment during hypercholanemic gestation significantly reduced fetal total serum bile acid levels due to a reduction in DCA, TDCA, and TCA compared with fetuses of untreated hypercholanemic mothers. Furthermore, analysis of fetal serum showed that OCA crosses the placenta and is present in the fetal compartment, predominantly as T-OCA. In line with this, hepatic *Cyp7a1* expression was reduced in fetuses from OCA-fed mice and further reduced in both CA and CA + OCA-fed groups. Interestingly, OCA treatment of hypercholanemic mothers was associated with an upregulation of placental transporters *Mrp2* (at the maternal-facing apical membrane) and *Oatp1b2* (basolateral membrane), which suggests enhanced elimination of fetal bile acids via the placenta. Increased placental expression of MRP2 has previously been associated with reduced bile acids in the fetal compartment in ICP pregnancies following UDCA treatment (3). Protein expression and bile acid transport studies would be required to confirm whether enhanced placental bile acid detoxification is responsible for this reduction in serum bile acids. The impact of OCA on fetal bile acid levels is of clinical interest due to the recent approval of OCA as a treatment for patients with PBC, as women with PBC may already be receiving OCA treatment when they become pregnant. In our study, we did not observe any detrimental effect of OCA on the fetus, in agreement with a previous study that found no impact on resorptions, number of fetuses, or fetal growth (10). However, detailed pathological investigations are required to assess the safety of fetal exposure to OCA.

In contrast to the fetus, maternal total serum bile acid levels were not reduced by OCA treatment. Furthermore, OCA treatment did not induce significant shifts in hepatic mRNA expression of bile acid homeostasis genes. These findings differ from a previous study of an estrogen-induced cholestasis rodent model reporting that OCA treatment induced bile flow and hepatocyte expression of *Shp*, *Bsep*, and *Mrp-2* while repressing *Ntcp* and *Cyp7a1* expression (15). A more recent study of estrogen-induced cholestasis in mice showed that OCA treatment did not upregulate mRNA expression of FXR targets in the liver or placenta but did increase hepatic FXR protein levels. Total serum bile acid levels were reduced in mothers; however, serum bile acids were only mildly elevated in this model (10). In contrast, a study investigating the effect of OCA administration to *Mdr2*^−/−^ mice found that dietary 0.03% OCA supplementation failed to exert any effect on bile flow and composition. This study further reported that both OCA and INT-767, a dual FXR and TGR5 agonist, were effective in reducing *Cyp7a1* and *Cyp8b1* gene expression, but only INT-767 administration resulted in increased hepatic *Shp* gene expression and BSEP protein expression (4). A possible explanation is that despite a far higher affinity of FXR for OCA, due to the activation of FXR by CA-feeding, this limited the impact of OCA in our study. This is perhaps surprising given that CA is a weak agonist of FXR [EC_50_ = 586 µM (25) in comparison with OCA (EC_50_ = 99nM (42)]. In line with this, CA has previously been shown to only partially induce BSEP in vitro in comparison with the natural FXR ligand CDCA (25). A possible explanation is the 10 times higher abundance of CA as compared with OCA, at least as measured in serum, which limited the impact of OCA. Regardless, OCA administration alone did not cause the expected robust upregulation of hepatic FXR targets. Of note is that OCA alone downregulated hepatic *Cyp7a1* expression, and this change was associated with an upregulation of *Shp* and *Fgf15* in the terminal ileum rather than hepatic *Shp* induction. Indeed, previous studies have demonstrated that OCA administration in rats leads to upregulation of *Shp* in the terminal ileum (46) and that, in mice lacking intestinal *Fxr*, OCA supplementation does not result in repression of hepatic *Cyp7a1* expression (50). Taken together with these studies, our findings suggest OCA acts primarily through ileal FXR to stimulate FGF15 secretion into the portal circulation and repress hepatic *Cyp7a1* expression in the maternal liver rather than via hepatic FXR to modulate the expression of other hepatic genes involved in bile acid homeostasis. Our study did not assess the effect of OCA on markers of liver damage. However, we are aware that CA feeding in twice the dose in male Swiss Albino mice has previously been shown to increase serum aspartate transaminase (AST), alanine transaminase (ALT), and alkaline phosphatase (AP) levels as well as hepatocyte size, mitosis, and necrosis (14).

Of note is that the expression of FXR target genes was decreased overall by pregnancy in both the liver and terminal ileum, which likely reflects the previously documented decreased gestational FXR activity (31, 33, 39). Nonetheless, in the liver of pregnant NC-fed females, OCA administration did not appear to efficiently overcome the reduction of FXR activity, and gene expression levels of FXR targets were similar. Conversely, in the maternal terminal ileum, the upregulation of *Shp* and *Fgf15* expression suggests an increase in FXR activity induced by OCA administration to NC-fed mice, but levels remained below those observed outside of pregnancy and so similarly indicate that OCA is unable to fully activate FXR in the terminal ileum. In support of this data, we also observed in a mouse model of gestational diabetes mellitus a diminished effect of OCA in pregnant mice compared with nonpregnant controls (30). This highlights the issue that limited efficacy of FXR agonists should be taken into account in treatment of pregnant women.

OCA was predominately unconjugated in the serum and the cecum, in contrast to mice fed OCA alone, where T-OCA predominated. This indicated a different pattern or activity of bile acid deconjugating microbiota. Indeed, 16S rRNA gene sequencing showed that there was an increase in relative abundance of *Bacteroidetes* and *Proteobacteria* (and also *Bacteroides* and *Bilophila* when analyzed at genus level) in the cecum of CA + OCA-fed females. We recently reported in pregnant mice that bile salt hydrolase, which was involved in deconjugation of bile acids, was exclusively detected in *Bacteroidetes*, with *Proteobacteria* also enriched in pregnancy, likely secondary to increased taurine made available after bile acid deconjugation (39). *Bilophila Wadsworthia* is known to be taurine metabolizing (24). These findings suggest that the predominance of unconjugated OCA in the serum of CA + OCA-fed mice could be due to an increase of *Bacteroidetes* and *Proteobacteria* in the gut.

OCA administration during hypercholanemic gestation did not reverse maternal dyslipidemia. Of note is that maternal OCA supplementation alone resulted in a decrease in serum total cholesterol due to a reduction in HDL cholesterol. A similar decrease in serum HDL cholesterol was seen in nonpregnant females. This decrease is not unexpected, as OCA has previously been shown to reduce HDL cholesterol in healthy humans, PBC, and NASH patients (22, 37, 43), and we recently reported that OCA reduced serum cholesterol in a mouse model of gestational diabetes mellitus (30). Furthermore, hepatic cholesterol content was raised in all bile acid-supplemented mice, albeit to a lesser extent in nonpregnant females fed an OCA diet. Dyslipidemia with hepatic cholesterol accumulation has previously been suggested to be associated with *Cyp7a1* repression found in cholestasis, as downregulation of bile acid synthesis from cholesterol leads to cholesterol accumulation in the liver (36, 48), suggesting that cholesterol accumulation in the liver may be proportional to hepatic *Cyp7a1* repression in our model.

Notably, serum triglycerides were reduced in pregnant mice that received OCA. This change is in line with previous studies showing that FXR activation reduces circulating triglycerides in *db/db* mice (52). Additionally, in patients with nonalcoholic fatty liver disease and type 2 diabetes, administration of 50 mg of OCA daily for 6 wk resulted in decreased serum triglyceride concentrations (34). However, OCA administration did not improve fetal dyslipidemia. In fact, maternal CA + OCA coadministration resulted in accumulation of cholesterol and FFAs in the fetal liver compared with fetuses of control mothers. Further investigations are needed to establish whether the upregulation of expression of placental lipid transporters *Abca1* and *Fatp4* may play a role.

In conclusion, OCA administration during hypercholanemic pregnancy, mimicking the raised serum bile acids observed in ICP, ameliorated fetal hypercholanemia, although maternal bile acid levels were not significantly decreased, and maternal and fetal dyslipidemia was not resolved. Significantly, no negative effects of maternal OCA treatment on maternal and fetal morphology, and most importantly, fetal survival, were observed. Because OCA may be used to treat women of reproductive age with PBC and NASH, further investigations into the safety of maternal and fetal exposure to OCA during pregnancy are warranted.

## GRANTS

The research was supported by the Wellcome Trust [092993/Z/10/Z], the Lauren Page Trust, and the Guy’s and St. Thomas’ Charity. Both J.R.M. and J.A.K.M., at Imperial College London, received financial support from the National Institute of Health Research (NIHR) Imperial Biomedical Research Centre (BRC) based at Imperial College Healthcare National Health Service (NHS) Trust and Imperial College London. C.W. is an NIHR Senior Investigator and receives support from the NIHR BRC based at Guy’s and St. Thomas’ NHS Foundation Trust and King’s College London.

The views expressed are those of the authors and not necessarily those of the NHS, the NIHR, or the Department of Health. According to Wellcome Trust’s Policy on data, software and materials management and sharing, all data supporting this study are available on request.

## DISCLOSURES

Intercept Pharmaceuticals provided the obeticholic acid used in the experiments described herein. L. Adorini and D. Shapiro are employees of Intercept Pharmaceuticals. H.-U. Marschall receives grants, personal fees, and nonfinancial support from Intercept Pharmaceuticals.

## AUTHOR CONTRIBUTIONS

V.P., G.P., and C.W. conceived and designed research; V.P., J.A.K.M., A.W., E.J., and H.-U.M. performed experiments; V.P., S.M., and J.A.K.M. analyzed data; V.P. and S.M. interpreted results of experiments; V.P., S.M., and J.A.K.M. prepared figures; V.P., S.M., and C.W. drafted manuscript; V.P., S.M., G.P., C.O., J.A.K.M., A.W., E.J., L.A., D.S., J.R.M., H.-U.M., and C.W. edited and revised manuscript; S.M. and C.W. approved final version of manuscript.

## References

[1] Abu-Hayyeh S, Ovadia C, Lieu T, Jensen DD, Chambers J, Dixon PH, Lövgren-Sandblom A, Bolier R, Tolenaars D, Kremer AE, Syngelaki A, Noori M, Williams D, Marin JJ, Monte MJ, Nicolaides KH, Beuers U, Oude-Elferink R, Seed PT, Chappell L, Marschall HU, Bunnett NW, Williamson C. Prognostic and mechanistic potential of progesterone sulfates in intrahepatic cholestasis of pregnancy and pruritus gravidarum. Hepatology 63: 1287–1298, 2016. doi:10.1002/hep.28265. 26426865 PMC4869673

[2] Ali AH, Carey EJ, Lindor KD. Recent advances in the development of farnesoid X receptor agonists. Ann Transl Med 3: 5, 2015. 25705637 10.3978/j.issn.2305-5839.2014.12.06 PMC4293481

[3] Azzaroli F, Mennone A, Feletti V, Simoni P, Baglivo E, Montagnani M, Rizzo N, Pelusi G, DE Aloysio D, Lodato F, Festi D, Colecchia A, Roda E, Boyer JL, Mazzella G. Clinical trial: modulation of human placental multidrug resistance proteins in cholestasis of pregnancy by ursodeoxycholic acid. Aliment Pharmacol Ther 26: 1139–1146, 2007. doi:10.1111/j.1365-2036.2007.03462.x. 17894656

[4] Baghdasaryan A, Claudel T, Gumhold J, Silbert D, Adorini L, Roda A, Vecchiotti S, Gonzalez FJ, Schoonjans K, Strazzabosco M, Fickert P, Trauner M. Dual farnesoid X receptor/TGR5 agonist INT-767 reduces liver injury in the Mdr2^−/−^ (Abcb4^−/−^) mouse cholangiopathy model by promoting biliary ${\text{HCO}}_{3}^{-}$ output. Hepatology 54: 1303–1312, 2011. doi:10.1002/hep.24537. 22006858 PMC3744065

[6] Baines SD, O’Connor R, Saxton K, Freeman J, Wilcox MH. Comparison of oritavancin versus vancomycin as treatments for clindamycin-induced Clostridium difficile PCR ribotype 027 infection in a human gut model. J Antimicrob Chemother 62: 1078–1085, 2008. doi:10.1093/jac/dkn358. 18772161

[7] Brouwers L, Koster MP, Page-Christiaens GC, Kemperman H, Boon J, Evers IM, Bogte A, Oudijk MA. Intrahepatic cholestasis of pregnancy: maternal and fetal outcomes associated with elevated bile acid levels. Am J Obstet Gynecol 212: 100.e1–100.e7, 2015. doi:10.1016/j.ajog.2014.07.026. 25046809

[8] Chappell LC, Bell JL, Smith A, Linsell L, Juszczak E, Dixon PH, Chambers J, Hunter R, Dorling J, Williamson C, Thornton JG, Ahmed I, Arya R, Beckett V, Bhide A, Brown H, Bugg G, Cameron H, Deole N, Dey M, Dwyer J, Fahel L, Gada R, Girling J, Haestier A, Hughes S, Indusekhar R, Jones B, Khan R, Kirkpatrick A, Knox E, Lincoln K, MacDougall M, Majoko F, McIntyre K, Noori M, Oakley W, Preston J, Ranka P, Rashid M, Salloum M, Samyraju M, Schram C, Sen S, Stone S, Tan B; PITCHES study group. Ursodeoxycholic acid versus placebo in women with intrahepatic cholestasis of pregnancy (PITCHES): a randomised controlled trial. Lancet 394: 849–860, 2019. doi:10.1016/S0140-6736(19)31270-X. 31378395 PMC6739598

[9] Chappell LC, Gurung V, Seed PT, Chambers J, Williamson C, Thornton JG; PITCH Study Consortium. Ursodeoxycholic acid versus placebo, and early term delivery versus expectant management, in women with intrahepatic cholestasis of pregnancy: semifactorial randomised clinical trial. BMJ 344: e3799, 2012. doi:10.1136/bmj.e3799. 22695903 PMC3420230

[10] Chen W, Gao XX, Ma L, Liu ZB, Li L, Wang H, Gao L, Xu DX, Chen YH. Obeticholic acid protects against gestational cholestasis-induced fetal intrauterine growth restriction in mice. Oxid Med Cell Longev 2019: 1–17, 2019. doi:10.1155/2019/7419249. 31827696 PMC6885290

[11] Conti-Ramsden F, McEwan M, Hill R, Wade J, Abraham G, Buckeldee O, Williamson C, Knight CL, Girling J, Chappell LC. Detection of additional abnormalities or co-morbidities in women with suspected intrahepatic cholestasis of pregnancy. Obstet Med 1753495X1986887, 2019. doi:10.1177/1753495X19868873. PMC7726172 33343695

[12] Dann AT, Kenyon AP, Wierzbicki AS, Seed PT, Shennan AH, Tribe RM. Plasma lipid profiles of women with intrahepatic cholestasis of pregnancy. Obstet Gynecol 107: 106–114, 2006. doi:10.1097/01.AOG.0000189096.94874.9c. 16394047

[13] Dixon PH, Williamson C. The pathophysiology of intrahepatic cholestasis of pregnancy. Clin Res Hepatol Gastroenterol 40: 141–153, 2016. doi:10.1016/j.clinre.2015.12.008. 26823041

[14] Fickert P, Zollner G, Fuchsbichler A, Stumptner C, Pojer C, Zenz R, Lammert F, Stieger B, Meier PJ, Zatloukal K, Denk H, Trauner M. Effects of ursodeoxycholic and cholic acid feeding on hepatocellular transporter expression in mouse liver. Gastroenterology 121: 170–183, 2001. doi:10.1053/gast.2001.25542. 11438506

[15] Fiorucci S, Clerici C, Antonelli E, Orlandi S, Goodwin B, Sadeghpour BM, Sabatino G, Russo G, Castellani D, Willson TM, Pruzanski M, Pellicciari R, Morelli A. Protective effects of 6-ethyl chenodeoxycholic acid, a farnesoid X receptor ligand, in estrogen-induced cholestasis. J Pharmacol Exp Ther 313: 604–612, 2005. doi:10.1124/jpet.104.079665. 15644430

[16] Freeman J, Baines SD, Jabes D, Wilcox MH. Comparison of the efficacy of ramoplanin and vancomycin in both in vitro and in vivo models of clindamycin-induced Clostridium difficile infection. J Antimicrob Chemother 56: 717–725, 2005. doi:10.1093/jac/dki321. 16143709

[17] Geenes V, Williamson C. Intrahepatic cholestasis of pregnancy. World J Gastroenterol 15: 2049–2066, 2009. doi:10.3748/wjg.15.2049. 19418576 PMC2678574

[18] Geenes V, Chambers J, Khurana R, Shemer EW, Sia W, Mandair D, Elias E, Marschall HU, Hague W, Williamson C. Rifampicin in the treatment of severe intrahepatic cholestasis of pregnancy. Eur J Obstet Gynecol Reprod Biol 189: 59–63, 2015. doi:10.1016/j.ejogrb.2015.03.020. 25864112

[19] Geenes V, Chappell LC, Seed PT, Steer PJ, Knight M, Williamson C. Association of severe intrahepatic cholestasis of pregnancy with adverse pregnancy outcomes: a prospective population-based case-control study. Hepatology 59: 1482–1491, 2014. doi:10.1002/hep.26617. 23857305 PMC4296226

[20] Geenes V, Lövgren-Sandblom A, Benthin L, Lawrance D, Chambers J, Gurung V, Thornton J, Chappell L, Khan E, Dixon P, Marschall HU, Williamson C. The reversed feto-maternal bile acid gradient in intrahepatic cholestasis of pregnancy is corrected by ursodeoxycholic acid. PLoS One 9: e83828, 2014. doi:10.1371/journal.pone.0083828. 24421907 PMC3885440

[21] Glantz A, Marschall HU, Mattsson LA. Intrahepatic cholestasis of pregnancy: Relationships between bile acid levels and fetal complication rates. Hepatology 40: 467–474, 2004. doi:10.1002/hep.20336. 15368452

[22] Hirschfield GM, Mason A, Luketic V, Lindor K, Gordon SC, Mayo M, Kowdley KV, Vincent C, Bodhenheimer HC Jr, Parés A, Trauner M, Marschall HU, Adorini L, Sciacca C, Beecher-Jones T, Castelloe E, Böhm O, Shapiro D. Efficacy of obeticholic acid in patients with primary biliary cirrhosis and inadequate response to ursodeoxycholic acid. Gastroenterology 148: P751–P761.E8, 2015. doi:10.1053/j.gastro.2014.12.005. 25500425

[23] Kong X, Kong Y, Zhang F, Wang T, Yan J. Evaluating the effectiveness and safety of ursodeoxycholic acid in treatment of intrahepatic cholestasis of pregnancy: a meta-analysis (a prisma-compliant study). Medicine (Baltimore) 95: e4949, 2016. doi:10.1097/MD.0000000000004949. 27749550 PMC5059052

[24] Laue H, Denger K, Cook AM. Taurine reduction in anaerobic respiration of Bilophila wadsworthia RZATAU. Appl Environ Microbiol 63: 2016–2021, 1997. doi:10.1128/AEM.63.5.2016-2021.1997. 9143131 PMC168491

[25] Lew JL, Zhao A, Yu J, Huang L, De Pedro N, Peláez F, Wright SD, Cui J. The farnesoid X receptor controls gene expression in a ligand- and promoter-selective fashion. J Biol Chem 279: 8856–8861, 2004. doi:10.1074/jbc.M306422200. 14684751

[26] Martineau M, Raker C, Powrie R, Williamson C. Intrahepatic cholestasis of pregnancy is associated with an increased risk of gestational diabetes. Eur J Obstet Gynecol Reprod Biol 176: 80–85, 2014. doi:10.1016/j.ejogrb.2013.12.037. 24462052

[27] Martineau MG, Raker C, Dixon PH, Chambers J, Machirori M, King NM, Hooks ML, Manoharan R, Chen K, Powrie R, Williamson C. The metabolic profile of intrahepatic cholestasis of pregnancy is associated with impaired glucose tolerance, dyslipidemia, and increased fetal growth. Diabetes Care 38: 243–248, 2015. doi:10.2337/dc14-2143. 25504029

[28] McDonald JAK, Mullish BH, Pechlivanis A, Liu Z, Brignardello J, Kao D, Holmes E, Li JV, Clarke TB, Thursz MR, Marchesi JR. Inhibiting growth of clostridioides difficile by restoring valerate, produced by the intestinal microbiota. Gastroenterology 155: 1495–1507.e15, 2018. doi:10.1053/j.gastro.2018.07.014. 30025704 PMC6347096

[29] McIlvride S, Dixon PH, Williamson C. Bile acids and gestation. Mol Aspects Med 56: 90–100, 2017. doi:10.1016/j.mam.2017.05.003. 28506676

[30] McIlvride S, Nikolova V, Fan HM, McDonald JAK, Wahlström A, Bellafante E, Jansen E, Adorini L, Shapiro D, Jones P, Marchesi JR, Marschall HU, Williamson C. Obeticholic acid ameliorates dyslipidemia but not glucose tolerance in mouse model of gestational diabetes. Am J Physiol Endocrinol Metab 317: E399–E410, 2019. doi:10.1152/ajpendo.00407.2018. 31237448 PMC6732461

[31] Milona A, Owen BM, Cobbold JF, Willemsen EC, Cox IJ, Boudjelal M, Cairns W, Schoonjans K, Taylor-Robinson SD, Klomp LW, Parker MG, White R, van Mil SW, Williamson C. Raised hepatic bile acid concentrations during pregnancy in mice are associated with reduced farnesoid X receptor function. Hepatology 52: 1341–1349, 2010. doi:10.1002/hep.23849. 20842631

[32] Milona A, Owen BM, van Mil S, Dormann D, Mataki C, Boudjelal M, Cairns W, Schoonjans K, Milligan S, Parker M, White R, Williamson C. The normal mechanisms of pregnancy-induced liver growth are not maintained in mice lacking the bile acid sensor Fxr. Am J Physiol Gastrointest Liver Physiol 298: G151–G158, 2010. doi:10.1152/ajpgi.00336.2009. 19815629 PMC2822506

[33] Moscovitz JE, Kong B, Buckley K, Buckley B, Guo GL, Aleksunes LM. Restoration of enterohepatic bile acid pathways in pregnant mice following short term activation of Fxr by GW4064. Toxicol Appl Pharmacol 310: 60–67, 2016. doi:10.1016/j.taap.2016.08.021. 27609522 PMC5064858

[34] Mudaliar S, Henry RR, Sanyal AJ, Morrow L, Marschall HU, Kipnes M, Adorini L, Sciacca CI, Clopton P, Castelloe E, Dillon P, Pruzanski M, Shapiro D. Efficacy and safety of the farnesoid X receptor agonist obeticholic acid in patients with type 2 diabetes and nonalcoholic fatty liver disease. Gastroenterology 145: 574–82.e1, 2013. doi:10.1053/j.gastro.2013.05.042. 23727264

[35] Mullish BH, Pechlivanis A, Barker GF, Thursz MR, Marchesi JR, McDonald JAK. Functional microbiomics: Evaluation of gut microbiota-bile acid metabolism interactions in health and disease. Methods 149: 49–58, 2018. doi:10.1016/j.ymeth.2018.04.028. 29704662 PMC6347095

[36] Murphy C, Parini P, Wang J, Björkhem I, Eggertsen G, Gåfvels M. Cholic acid as key regulator of cholesterol synthesis, intestinal absorption and hepatic storage in mice. Biochim Biophys Acta 1735: 167–175, 2005. doi:10.1016/j.bbalip.2005.06.001. 15994119

[37] Neuschwander-Tetri BA, Loomba R, Sanyal AJ, Lavine JE, Van Natta ML, Abdelmalek MF, Chalasani N, Dasarathy S, Diehl AM, Hameed B, Kowdley KV, McCullough A, Terrault N, Clark JM, Tonascia J, Brunt EM, Kleiner DE, Doo E; NASH Clinical Research Network. Farnesoid X nuclear receptor ligand obeticholic acid for non-cirrhotic, non-alcoholic steatohepatitis (FLINT): a multicentre, randomised, placebo-controlled trial. Lancet 385: 956–965, 2015. doi:10.1016/S0140-6736(14)61933-4. 25468160 PMC4447192

[38] Nikolova V, Papacleovoulou G, Bellafante E, Borges Manna L, Jansen E, Baron S, Abu-Hayyeh S, Parker M, Williamson C. Changes in LXR signaling influence early-pregnancy lipogenesis and protect against dysregulated fetoplacental lipid homeostasis. Am J Physiol Endocrinol Metab 313: E463–E472, 2017. doi:10.1152/ajpendo.00449.2016. 28420650 PMC5689017

[39] Ovadia C, Perdones-Montero A, Spagou K, Smith A, Sarafian MH, Gomez-Romero M, Bellafante E, Clarke LCD, Sadiq F, Nikolova V, Mitchell A, Dixon PH, Santa-Pinter N, Wahlström A, Abu-Hayyeh S, Walters JRF, Marschall HU, Holmes E, Marchesi JR, Williamson C. Enhanced microbial bile acid deconjugation and impaired ileal uptake in pregnancy repress intestinal regulation of bile acid synthesis. Hepatology 70: 276–293, 2019. doi:10.1002/hep.30661. 30983011 PMC6619257

[40] Ovadia C, Seed PT, Sklavounos A, Geenes V, Di Ilio C, Chambers J, Kohari K, Bacq Y, Bozkurt N, Brun-Furrer R, Bull L, Estiú MC, Grymowicz M, Gunaydin B, Hague WM, Haslinger C, Hu Y, Kawakita T, Kebapcilar AG, Kebapcilar L, Kondrackienė J, Koster MPH, Kowalska-Kańka A, Kupčinskas L, Lee RH, Locatelli A, Macias RIR, Marschall HU, Oudijk MA, Raz Y, Rimon E, Shan D, Shao Y, Tribe R, Tripodi V, Yayla Abide C, Yenidede I, Thornton JG, Chappell LC, Williamson C. Association of adverse perinatal outcomes of intrahepatic cholestasis of pregnancy with biochemical markers: results of aggregate and individual patient data meta-analyses. Lancet 393: 899–909, 2019. doi:10.1016/S0140-6736(18)31877-4. 30773280 PMC6396441

[41] Papacleovoulou G, Abu-Hayyeh S, Nikolopoulou E, Briz O, Owen BM, Nikolova V, Ovadia C, Huang X, Vaarasmaki M, Baumann M, Jansen E, Albrecht C, Jarvelin MR, Marin JJ, Knisely AS, Williamson C. Maternal cholestasis during pregnancy programs metabolic disease in offspring. J Clin Invest 123: 3172–3181, 2013. doi:10.1172/JCI68927. 23934127 PMC3696570

[42] Pellicciari R, Fiorucci S, Camaioni E, Clerici C, Costantino G, Maloney PR, Morelli A, Parks DJ, Willson TM. 6alpha-ethyl-chenodeoxycholic acid (6-ECDCA), a potent and selective FXR agonist endowed with anticholestatic activity. J Med Chem 45: 3569–3572, 2002. doi:10.1021/jm025529g. 12166927

[43] Pencek R, Marmon T, Roth JD, Liberman A, Hooshmand-Rad R, Young MA. Effects of obeticholic acid on lipoprotein metabolism in healthy volunteers. Diabetes Obes Metab 18: 936–940, 2016. doi:10.1111/dom.12681. 27109453

[44] Pusl T, Beuers U. Intrahepatic cholestasis of pregnancy. Orphanet J Rare Dis 2: 26, 2007. doi:10.1186/1750-1172-2-26. 17535422 PMC1891276

[45] Tremaroli V, Karlsson F, Werling M, Ståhlman M, Kovatcheva-Datchary P, Olbers T, Fändriks L, le Roux CW, Nielsen J, Bäckhed F. Roux-en-Y gastric bypass and vertical banded gastroplasty induce long-term changes on the human gut microbiome contributing to fat mass regulation. Cell Metab 22: 228–238, 2015. doi:10.1016/j.cmet.2015.07.009. 26244932 PMC4537510

[46] Úbeda M, Lario M, Muñoz L, Borrero MJ, Rodríguez-Serrano M, Sánchez-Díaz AM, Del Campo R, Lledó L, Pastor Ó, García-Bermejo L, Díaz D, Álvarez-Mon M, Albillos A. Obeticholic acid reduces bacterial translocation and inhibits intestinal inflammation in cirrhotic rats. J Hepatol 64: 1049–1057, 2016. doi:10.1016/j.jhep.2015.12.010. 26723896

[47] Van den Abbeele P, Grootaert C, Marzorati M, Possemiers S, Verstraete W, Gérard P, Rabot S, Bruneau A, El Aidy S, Derrien M, Zoetendal E, Kleerebezem M, Smidt H, Van de Wiele T. Microbial community development in a dynamic gut model is reproducible, colon region specific, and selective for Bacteroidetes and Clostridium cluster IX. Appl Environ Microbiol 76: 5237–5246, 2010. doi:10.1128/AEM.00759-10. 20562281 PMC2916472

[48] Wang L, Han Y, Kim CS, Lee YK, Moore DD. Resistance of SHP-null mice to bile acid-induced liver damage. J Biol Chem 278: 44475–44481, 2003. doi:10.1074/jbc.M305258200. 12933814

[49] Wikström Shemer E, Marschall HU, Ludvigsson JF, Stephansson O. Intrahepatic cholestasis of pregnancy and associated adverse pregnancy and fetal outcomes: a 12-year population-based cohort study. BJOG 120: 717–723, 2013. doi:10.1111/1471-0528.12174. 23418899

[50] Xu Y, Li F, Zalzala M, Xu J, Gonzalez FJ, Adorini L, Lee YK, Yin L, Zhang Y. Farnesoid X receptor activation increases reverse cholesterol transport by modulating bile acid composition and cholesterol absorption in mice. Hepatology 64: 1072–1085, 2016. doi:10.1002/hep.28712. 27359351 PMC5033696

[51] Zhang Y, Jackson JP, St Claire RL III, Freeman K, Brouwer KR, Edwards JE. Obeticholic acid, a selective farnesoid X receptor agonist, regulates bile acid homeostasis in sandwich-cultured human hepatocytes. Pharmacol Res Perspect 5: e00329, 2017. doi:10.1002/prp2.329. 28805978 PMC5684861

[52] Zhang Y, Lee FY, Barrera G, Lee H, Vales C, Gonzalez FJ, Willson TM, Edwards PA. Activation of the nuclear receptor FXR improves hyperglycemia and hyperlipidemia in diabetic mice. Proc Natl Acad Sci USA 103: 1006–1011, 2006. doi:10.1073/pnas.0506982103. 16410358 PMC1347977

